# Micro‐bacterial assessment of disposable gowns with a focus on green endoscopy in gastrointestinal endoscopy procedures: A Japanese pilot study for healthcare waste reduction

**DOI:** 10.1002/deo2.70016

**Published:** 2024-09-24

**Authors:** Sakiko Naito, Itaru Nakamura, Takahiro Muramatsu, Yasuyuki Kagawa, Masakatsu Fukuzawa, Takao Itoi

**Affiliations:** ^1^ Department of Gastroenterology and Hepatology Tokyo Medical University Tokyo Japan; ^2^ Department of Infection Control and Prevention Tokyo Medical University Tokyo Japan

**Keywords:** bacteria, endoscopy, green endoscopy, personal protective equipment, sustainable development goals

## Abstract

**Objective:**

This study aimed to implement green endoscopy through the microbiological assessment of gowning techniques during endoscopy to reduce carbon emissions and separate medical waste.

**Methods:**

Twenty‐five patients who performed esophagogastroduodenoscopy from March to May 2024 were included in this study. Four sections of the isolation gowns (anterior, posterior, right, and left) were cut into 2 cm^2^ after endoscopy, and the rate of microbial contamination was examined using the stamp method.

**Results:**

The endoscopic examination time was 8 min (6−12), and endoscopy was performed by 10 expert endoscopists, six endoscopists, and nine residents. The overall isolation gown contamination rate was 56%, with 25%, 20.8%, 20.8%, and 33.3% in the front, back, as well as right and left arms, respectively. The rates of isolation gown contamination rates in the expert endoscopists, endoscopists, and residents groups were 30%, 50%, and 77.8%, respectively, with a higher rate in the residents group. Regardless of the physician's performance, bacterial detection was consistently higher in the left arm (42.9% vs. 40% vs. 25%; *p* = 0.093). The detected bacteria comprised 58% Gram‐positive and 42% Gram‐negative organisms, including those from tap water used for endoscopy bacteria and obtained from the participant's skin or mouth. No pathogenic organisms were detected.

**Conclusions:**

The bacteria detected in disposable gowns after gastrointestinal endoscopy were non‐pathogenic. Thus, our findings suggest that changing all personal protective equipment of respective endoscopes might not be essential. We advocate for green endoscopy to achieve sustainable development goals and reduce medical waste.

## INTRODUCTION

In Western countries, recommendations for waste management and recycling through reuse have recently been widely advocated to enable the realization of sustainable development goals (SDGs) and green endoscopy has been introduced and promoted in endoscopy‐related procedures by measuring and assessing carbon dioxide emissions.^1^, ^2^ The Green Endoscopy Initiative was introduced and promoted to measure and assess carbon dioxide emissions in endoscopy‐related procedures. Disposable duodenoscopes are recommended for infection control, especially for duodenoscopes used in cholangiopancreatography, and can be used in practice. However, recycling through reuse and appropriate waste management are recommended to reduce CO_2_ emissions. It has been reported that 56% of upper gastrointestinal (GI) endoscopies and 23%−52% of lower GI endoscopies are performed inappropriately, as the diagnostic rate is not improved by examining indeterminate symptoms, and reduction of unnecessary endoscopies is encouraged using the 3Rs (reduce, reuse, and recycle).^3^, ^4^ Such environmentally sustainable initiatives and environmentally friendly endoscopic treatment strategies are essential for developing safer endoscopic‐related procedures in the future.

The coronavirus disease 2019 (COVID‐19) pandemic significantly impacted endoscopy. To ensure infection prevention, personal protective equipment (PPE), masks, and eye guards were used per individual patient, which has increased medical waste. Although evidence has shown that PPE is effective in reducing transmission of the severe acute respiratory syndrome coronavirus 2 (SARS‐CoV‐2) virus, the need and extent of using PPE in different settings remains controversial.^5^ Although environmental measures are needed to prevent viral spread, recommendations for appropriate case‐specific gowning techniques could lead to sustainable environmental protection, and cultures of microorganisms and bacteria on gowns before and after endoscopy may provide information on environmental exposure. In order to achieve the SDGs with a focus on green endoscopy, which is attracting increasing attention and being implemented globally, it is necessary to separate medical waste in endoscopy‐related procedures and implement initiatives that lead to feasible cost‐effectiveness considering greenhouse gas (GHG) emissions. In this study, we evaluated and examined whether exposure to infection occurs during endoscopy, what kind of bacterial exposure actually occurs, as well as whether the bacteria cause environmental contamination and associated exposure to infection by culturing bacteria attached to PPE used during endoscopy.

Therefore, with a focus on green endoscopy, this study aimed to conduct a microbiological assessment of gowning techniques during endoscopy to reduce carbon emissions and separate medical waste.

## METHODS

### Participants and design

This single‐center prospective pilot study included isolation gowns from 25 cases obtained to identify microorganisms after diagnostic esophagogastroduodenoscopy from January to March 2024 at Tokyo Medical University Hospital which has 900 beds in Shinjuku, Tokyo, Japan. Current clinical investigations were performed during routine endoscopic screening or surveillance.

### Identification methods for micro‐organisms on gowns after endoscopy

The isolation gowns used during the examination, body polypropylene non‐woven cuff polyester (MEDLINE Inc.), were cut into 2 cm squares and applied to a blood agar medium for bacterial identification. Samples were collected from four locations per gown, including the front and back of the gown and both arms, after endoscopy. The extent of contamination during the examination was evaluated by comparing the microorganisms on the gown before and after esophagogastroduodenoscopy. Bacteria were classified as oral resident or opportunistic bacteria.

### Microbiological testing methods

We examined the detection rate and breakdown of microorganisms on the 2‐cm cut isolation gowns using the food stamp method. The microorganisms on the gowns were attached to the Food Stamp ‘Nissui’ standard agar medium (Nissui Pharmaceutical Co., Ltd.). The medium was heaped, dispensed, and coagulated in a special petri dish with an area of 10 cm^2^. Collection and application of specimens were completed by pressing the capped medium directly onto the surface of the specimens and cultivated at 35°C for 72 h. If bacteria were detected at 24 and 48 h, they were applied to a blood agar medium and analysis was performed using mass spectrometry to identify the bacteria.

### Procedures for endoscopy

Two expert endoscopists (Sakiko Naito and Masakatsu Fukuzawa), two endoscopists, and two residents examined the endoscopy time, microbiological detection rate, and extent of contamination. An expert endoscopist is defined as a physician licensed as a Japan Gastroenterological Endoscopy Society (JGES)‐certified endoscopy instructor. An endoscopist is defined as a physician licensed as a JGES‐certified endoscopy specialist, and a resident is defined as a resident following endoscopy training (trainees). All patients underwent oral endoscopy without using surgical masks to cover the patient, boxes, plastic enclosures, or “endoscopic shields”. Endoscopy was performed using an Olympus GIF‐H290Z or GIF‐XZ1200 (Olympus Medical Systems) endoscope. Sedation endoscopy was performed under midazolam or midazolam with pethidine.

### Statistical analysis

The median and interquartile range (IQR) were calculated for endoscopic procedure time. The contamination rate of the isolation gown was analyzed using Fisher's exact or chi‐squared tests, and continuous variables were analyzed using the Mann−Whitney U test. Significant differences in parameters among the three endoscopic treatment groups were determined using the Kruskal−Wallis test. All statistical analyses were performed using SPSS software (version 29; IBM Japan), and statistical significance was set at *p* < 0.05.

## RESULTS

### Extent of contamination during endoscopy

In this study, we examined 25 cases and 100 specimens. A total of 10, six, and nine endoscopic examinations were performed by experts, endoscopists, and residents, respectively (Tables 1 and 2). The total time to perform the endoscopy was 8 minutes. In total, 11 (44%) sedation endoscopic examinations were performed, and the contamination rate isolation gown was 56%, with 24% in the front, 20% in the back, 20% in the right arm, and 32% in the left arm. Comparing physicians performing the procedure, the median endoscopy time was 7, 6, and 11 min for experts, endoscopists, and residents, respectively, without significant differences. The median endoscopy time under sedation tended to be longer among residents (50% vs. 16.7% vs. 55.6%), but this was not significant. In a comparison of isolation gowns contamination rate of the isolation gown in endoscopies performed by experts, endoscopists, and residents was 40%, 50%, and 77.8%, respectively. Additionally, when compared by part, the contamination rates of the isolation gown in endoscopies performed by experts, endoscopists, and residents were 28.5%, 20%, and 25% in the front; 14.3%, 0%, and 33.3% in the back; 14.3%, 40%, and 16.7% in the right arm; as well as 42.9%, 40%, and 25% in the left arm, respectively. The rates of detecting microorganisms on isolation gowns worn and not worn without undergoing endoscopy were also examined. Bacteria were detected in one location (25%) on the gowns worn, which were one of the commensal bacteria (kocuria palustris). No bacteria were detected in the isolation gowns not worn (Table S1).

**TABLE 1:** Endoscopic examination components.

Examination procedure by physicians (Expert endoscopist/endoscopist/resident)	10/6/9
Endoscopic procedure time, min, median, (IQR)	8 (6−12)
Number of endoscopic examinations Sedation/Non‐sedation, n (%)	11 (44) 14 (56)
Isolation gown contaminate rate, %	56
Detail of isolation gown contaminate rate: front/back/right/left, %	24/20/20/32

Abbreviation: IQR, interquartile range.

**TABLE 2:** Details of endoscopy by each group.

	Expert endoscopist	Endoscopist	Resident	*p*‐value
Endoscopic procedure time, minute, median, (IQR)	7 (4−11)	6 (5−9)	11 (8−14)	0.605
Number of endoscopic examination with sedation, *n* (%)	5 (50)	1 (16.7)	5 (55.6)	0.637
Isolation gown contaminate rate, %	40	50	77.8	0.102
Detail of isolation gown contaminate rate: front/back/right/left, %	28.5/14.3/14.3/42.9	20/ 0/40/40	25/33.3/16.7/25	0.093

AbbreviationL IQR, interquartile range.

There was a trend towards slightly longer examination times taken by residents (7 and 6 min for non‐residents and 11 min for residents), but this was not significant. The contamination rate of the isolation gown in endoscopies performed by experts, endoscopists, and residents was 40%, 50%, and 77.8%, respectively, with residents having a higher contamination rate but not significant (*p* = 0.102). A high rate of contamination was found in the left arm in all procedures (42.9% vs. 40% vs. 25%; *p* = 0.093).

### Details of microbial composition

The bacteria identified were non‐pathogenic opportunistic infections and oral bacteria.

**FIGURE 1:**
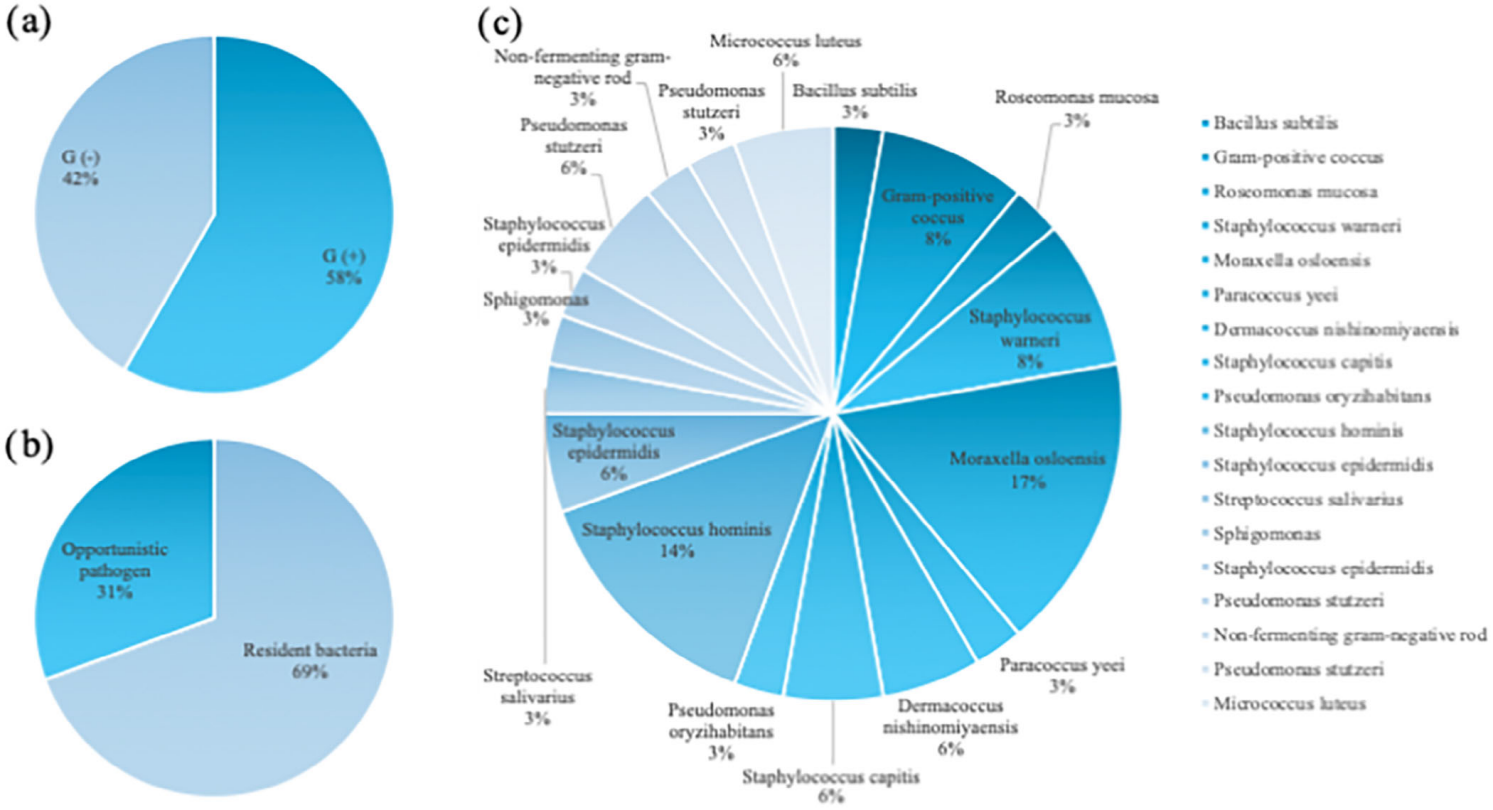
Description of microorganisms detected after endoscopic examination. (a) Details of gram‐negative (G(−)) and positive (G(+)) bacteria. There was no difference in detection rates. (b) Breakdown of opportunistic pathogens and resident bacteria. (c) Details of bacteria detected on gowns.auth‐info.

The detected bacteria, including coagulase‐negative *Staphylococcus*, were 58% Gram‐positive and 42% Gram‐negative bacteria. In addition, In addition, and 69% of oral resident bacteria and 31% of opportunistic bacteria from the tap water used for endoscopy. (Figure 1).

## DISCUSSION

In this study, we examined whether and to what extent isolation gowns were contaminated by splashed microorganisms during esophagogastroduodenoscopy and determined the environmental contamination rate. We obtained a 56% microbial contamination rate. The microorganisms comprised oral and oral resident bacteria, whereas the splashed bacteria were opportunistic bacteria or from non‐pathogenic tap water used for washing.^6^ In this study, we aimed to investigate green endoscopy for CO_2_ reduction. Therefore, we examined the approach to reducing CO_2_ by examining what kind of microorganisms are exposed to during endoscopy and detecting bacteria, based on scientific evidence. As a result, no bacteria that could be a source of infection were detected, and thus we detected bacteria produced by oral commensal bacteria and tap water only. This suggests that wearing PPE in selected cases is not necessary, leading to green endoscopy. Although gown contamination was associated with the examination procedure, no potentially hazardous microorganisms were detected. To our knowledge, this is the first study to report an actual microbial culture to assess contamination required for gowning techniques to ensure green endoscopy in reducing medical waste in view of the SDGs and CO_2_ emissions.

The new concept, ‘‘green endoscopy,’ has recently emerged, as efforts to promote sustainability and reduce carbon emissions at the international level are currently being explored.^7^, ^8^ The healthcare system contributes the largest amount of GHG emissions, accounting for 13% of total emissions.^9^ Of these, GI endoscopy is considered one of the most common sources of medical waste and the third largest source of hazardous waste, with an estimated 3.1 kg of waste generated per procedure.^10^, ^11^ For every 100 GI endoscopy procedures, 303 kg of solid waste and 1385 gallons of liquid waste are generated, with a carbon footprint of 1501 kg requiring 1.8 acres of forest to absorb and 20% recycling.^11^ For the SDG to be achieved, reducing CO_2_ emissions is of paramount importance.^12^, ^13^

### Recommendations for reducing CO_2_ emissions in endoscopic procedures

In the 21st century, climate change and the destruction of ecosystems by humans have become the most significant challenges requiring urgent action. The American Society for Gastrointestinal Endoscopy^1^ reports that more than 8 million deaths each year are due to air pollution, which is approximately 10 times the mortality rate for colorectal cancer. Endoscopy‐related procedures are a major source of carbon emissions in the healthcare sector, requiring efforts towards decarbonization.^6^ The European Society of Gastrointestinal Endoscopy and European Society of Gastroenterology and Endoscopy Nurses and Associates^2^ also report that climate change is a major threat, emphasizing the importance of appropriate endoscopic practices and rational use of disposable accessories. Medical waste from medical treatment activities is also a major contributor, with endoscopy‐related procedures having the greatest influence.^10^, ^14^ It has been reported that 18 million endoscopies are performed in the USA and 1.5 kg of medical waste is generated from a single endoscopic procedure.^15^ A net‐zero GHG‐emitting healthcare system is recommended by 2022, with recommendations to reduce unnecessary procedures by introducing 3R programs, as well as quantifying and minimizing the environmental impact of GI endoscopy.^16^ Although endoscopy is important for early detection and early treatment, it should be performed appropriately for the disease state, and appropriate examinations can be cost‐effective. We provided scientific evidence that droplets had no impact on environmental contamination, suggesting that gowning techniques with PPE are not always necessary and should be used depending on the case. If isolation gowns do not need to be changed after each examination, the amount of medical waste generated can be greatly reduced, encouraging green endoscopy. However, even though environmental pollution is a global problem, guidance on the impact of medical practice on the environment is not evaluated or provided in the medical field currently, which is a major problem.

### Prevention of infection exposure due to differences in background factors

Oral bacteria were detected in 78.1% of the cases. Sedated endoscopy was performed in 16% and 57.1% of the tests where oral and opportunistic bacteria were detected, respectively. There was no difference in bacterial detection between tests. The left arm was closer to the patient's side and thus more susceptible to exposure, suggesting that the 20% of bacteria detected on the back were indigenous to the examiner due to PPE desorption. The bacterial culture showed indigenous bacteria on the left arm of the PPE worn without performing endoscopy. There was no difference in the breakdown of bacteria between opportunistic (55.6%) and oral commensal (44.4%). It is possible that the PPE was removed first without removing the gloves during PPE removal after endoscopy. The procedure of removing the gloves first and then the gown after the examination may have been followed more frequently by residents because they removed the PPE with the gloves on. Thorough infection control measures should be taken regarding the order of PPE removal and putting on to prevent contamination of the surrounding environment. Bacterial exposure did not differ depending on whether the patient was sedated or not and was particularly likely to occur in residents because of the longer examination and exposure time. Based on these results, measures to prevent environmental exposure are necessary, and guidance for residents on environmental measures and infection control through careful hand hygiene should be practiced.

### Effects of infection transmission on environmental pollution

The COVID‐19 pandemic led to a significant increase in the use of PPE during endoscopy in order to prevent the effect of aerosol generation on endoscopy staff and the environment. According to Rizan et al., the carbon footprint of PPE was 106.478 tons of CO_2_e in 6 months during COVID‐19, indicating the significant environmental impact of PPE and the importance of waste management.^5^, ^17^ We have reported that COVID‐19 affects older patients and those suffering from malignant tumors, who are easily infected. The American Society for Gastrointestinal Endoscopy guidelines generally recommend the use of PPE during endoscopy because of the possibility of exposure to unknown viruses or bacteria and report the importance of cleaning endoscopic instruments.^18^, ^19^ In the present study, exposure to bacteria suggestive of infection was not detected even in patients with a strong vomiting reflex who were considered at high risk of infection, and the endoscopists were not affected by exposure to infection. In cases such as COVID‐19 and tuberculosis where splash infection is suggested, or in cases where sufficient interviewing is not possible, such as in emergency endoscopy, taking measures to prevent infection using PPE may be necessary. However, in cases where there is no risk of splash infection during endoscopic examinations, PPE may not be necessary. In all cases, endoscopes should be cleaned according to guidelines to prevent infection and infection control measures should be taken on a patient‐by‐case basis. Since this is a study with a small number of cases, it is possible that we only considered exposure to a small portion of bacteria. Conducting a study with a large number of cases in consideration of the results of this study to further establish the scientific basis is necessary, including the frequency of using isolation gowns in a consistent method. In the present study,^20^ it is also considered that microorganisms on gowns have almost zero chance of becoming pathogenic, and it is inferred that by selecting the appropriate cases, it is possible to reduce the impact of environmental contamination to zero without infection control precautions using PPE.

Preventing exposure to environmental infection through careful hand hygiene at each examination is possible as microorganisms adhering to the gown were identified as commensal or opportunistic bacteria, which are usually not transmitted. As discussed above, wearing isolation gowns is unnecessary in many cases, allowing for environmentally friendly medical care. Therefore, strategies are needed to reduce CO_2_ emissions in endoscopy and establish standards for not only facilities but also the whole system to reduce CO_2_ emissions, making endoscopy an environmentally sustainable model and enabling the realization of SDGs.

This study has several limitations. Although the microbial contamination rate of the isolation gown was examined using the stamp method for assessing the contamination rates, it is possible that the endoscopists' epidermal *Staphylococci* were what was detected, although micro‐organisms were not frequently detected on the front and back of the isolation gowns. Second, the number of isolation gowns discharged in a day was not calculated; therefore, daily medical waste calculation was not possible. Third, this is a study with a small number of cases and only some bacterial exposures were examined. Conducting a study examining a large number of cases considering the results of this study and further substantiating the scientific evidence, including the frequency of continuous use of isolation gowns, is necessary. Finally, only esophagogastroduodenoscopy was considered and other GI endoscopy procedures were not included, which might limit the generalizability of our findings, and should be assessed in future studies.

In conclusion, the bacteria detected in disposable gowns after GI endoscopy were mainly non‐pathogenic organisms, and changing all PPE might not be essential. We recommend a green endoscopy technique that achieves the SDGs and reduces medical waste.

## CONFLICT OF INTEREST STATEMENT

The author Takao Itoi is the editor‐in‐chief of Digestive Endoscopy Open, and the other authors declare no conflict of interest for this article.

## ETHICS STATEMENT

This study was approved by the Medical Ethics Review Committee of Tokyo Medical University Hospital between March and May 2024 (registration number: TS2023‐0695). The study protocol conformed to the ethical guidelines of the Declaration of Helsinki 1964 and was approved by the Institutional Human Research Committee.

## PATIENT CONSENT STATEMENT

N/A

## Supporting information



Supplemental Table 1. Microbiological culture of PPE without Endoscopy
