# Supplementary material for: Micro‐bacterial assessment of disposable gowns with a focus on green endoscopy in gastrointestinal endoscopy procedures: A Japanese pilot study for healthcare waste reduction
Source: DEN Open. 2024 Sep 24;5(1):e70016. doi: 10.1002/deo2.70016 (PMC11422662; doi:10.1002/deo2.70016)
Supplement: Supplementary file 1 — Supplemental Table 1. Microbiological culture of PPE without Endoscopy [file DEO2-5-e70016-s001.docx]

| Supplemental Table 1. Microbiological culture of PPE without Endoscopy | | |
| --- | --- | --- |
|  | Unclothed PPE | Clothed PPE |
| Front | - | - |
| Back | - | - |
| Right | - | - |
| Left | - | Resident bacteria (*kocuria palustris*) |

PPE, personal protective equipment.
